# A birth cohort study in the Middle East: the Qatari birth cohort study (QBiC) phase I

**DOI:** 10.1186/s12889-017-4848-9

**Published:** 2017-10-23

**Authors:** Eman Sadoun, Vasiliki Leventakou, Maribel Casas, Heba Fawzy Ahmed, Manolis Kogevinas, Eleni Fthenou

**Affiliations:** 1 Health Care Quality Management and Patient Safety Department, Ministry of Public Health-Qatar, 42, Doha, Qatar; 2 Barcelona Institute for Global Health (ISGlobal), Barcelona, Spain; 3 IMIM (Hospital del Mar Medical Research Institute), Barcelona, Spain; 4 Qatar Biobank for Medical Research, Qatar Foundation, Building 17, Hamad Medical city, P.O. Box 5825, Doha, Qatar

**Keywords:** Birth cohort, Pregnancy, Recruitment, Mother-child

## Abstract

**Background:**

The latest scientific reports raise concerns about the rapidly increasing burden of chronic diseases in the state of Qatar. Pregnant Qatari women often confront complications during pregnancy including gestational diabetes, hypertension, abortion and stillbirth. The investigation of early life environmental, genetic, nutritional and social factors that may affect lifelong health is of great importance. Birth cohort studies offer a great opportunity to address early life hazards and their possible long lasting effects on health.

**Methods/design:**

The Qatari Birth Cohort study is the first mother-child cohort study in the Middle East Area that aims to assess the synergetic role of environmental exposure and genetic factors in the development of chronic disease and monitor woman and child health and/or obstetric characteristics with high prevalence. The present manuscript describes the recruitment phase of the study (duration: 2 years; expected number: 3000 families), where the pregnant Qatari women and their husbands are being contacted before the 15th week of gestation at the Primary Health Care Centers. The consented participants are interviewed to obtain information on several factors (sociodemographic characteristics, dietary habits, occupational/environmental exposure) and maternal characteristics are assessed based on anthropometric measurements, spirometry, and blood pressure. Pregnant women are invited to provide biological samples (blood and urine) in each trimester of their pregnancy, as well as cord blood at delivery. Fathers are also asked to provide biological samples.

**Discussion:**

The present study provides invaluable insights into a wide range of early life factors affecting human health. With a geographical focus on the Middle East, it will be a resource for information to the wider scientific community and will allow the formulation of effective policies with a primary focus on public health interventions for maternal and child health.

## Background

Early life events during critical developmental periods, may have significant impacts on the maintenance of lifelong healthiness. Recent epidemiological and experimental evidence points out how an adverse intrauterine environment, related to maternal dietary or placental insufficiency, could program susceptibility of the fetus to later development of cardiovascular or metabolic diseases such as obesity, hypertension, insulin resistance and type 2 diabetes [1, 2]. The ‘early life programming’ concept was originally proposed so as to explain the link between maternal nutritional deficits during pregnancy, low birth weight (less than 2.500 g) and risk for disease in lifespan [3]. This concept has been later expanded to the developmental origins of health and disease (DOHaD) model, which links fetal development to an unfavorable environment with the development of non-communicable diseases in later life [4, 5]. Psychiatric research has been inspired by the DOHaD model with a key focus being to provide insights into the etiology of mental disorders emerging from early life traits. Pregnancy and birth cohort studies provide the most suitable design to assess early life hazards at critical time points during development, and their possible long-lasting effects.

The rising incidence of chronic disease constitutes a major public health issue affecting many countries worldwide. In the Middle East, recent reports have raised this concern for member states of the Gulf Cooperative Council (GCC), where a higher burden of chronic diseases than most developed countries is observed [6–8]. The World Health Organization (WHO) Qatar STEPS survey of chronic disease risk factors in 2012 reported that almost half of the general Qatari population is at high risk for developing chronic diseases [9]. High prevalence rates of diabetes [10], cardiovascular disease [11], metabolic syndrome [12], hypertension [13], and mental illness have been reported in the population of Qatar [14]. Given the high levels of obesity and diabetes in Qatar, it is likely that a significant percentage of pregnancy complications will be associated with maternal obesity, type 2 diabetes and gestational diabetes. There is also a high concern on the increased incidence of mental disorders (such as postpartum depression, anxiety, and personality disorder) in Qatari women at the reproductive age [15].

In line with the increased incidence of chronic diseases in Qatar, there also exists a higher than expected prevalence of specific adverse birth outcomes such as stillbirths (6.9%), preterm births (8.5%) and low birth weight (8.8%) deliveries [16, 17]. Recent studies have reported that the impact of stillbirths on Qatar’s perinatal mortality has increased 17% within two decades [16], but with no sufficient data explaining this incidence. Moreover, gestational diabetes and gestational hypertension are the main complications observed in Qatari pregnant women [18].

Given the limited number of birth cohort studies in the Middle East, which mainly focus on specific health outcomes, the establishment of a mother-child cohort in Qatar is an excellent opportunity to address a broad range of research questions. The Qatari Birth Cohort Study (QBiC) aims to examine how environmental, genetic, nutritional and social factors affect health at first stages of life and childhood. The study focuses on the two most critical time windows of human development, the in utero exposure of the fetus through maternal exposures and the early life exposures as they are related to transgenerational alterations in ‘omic’ profile and disease onset. This project will comprise risk and benefit analyses including maternal and early life health monitoring as well as an extensive range of ‘omic’ analyses for the in depth investigation of the impact of genome-exposure synergy in the establishment of adverse birth outcomes and chronic diseases development. Moreover, the present study will investigate the increasing stillbirth rates [16, 17] and the concern of high abortion that was raised by the Permanent Population Committee at Qatar Population Status 2012 report [19]. The envisaged work has major potential for translational exploitation both national and international aiming to develop harmonized data for future collaborations with international birth cohorts. The purpose of the present protocol is to introduce the study design and the methods that are used for the recruitment phase of this longitudinal birth cohort in the state of Qatar.

## Methods/design

The QBiC study is the first mother-child cohort in the state of Qatar that aims to recruit and follow up for 5 years a sample of 3000 pregnant women and their children from birth to childhood. The main objectives of the cohort are briefly cited below:

- To evaluate maternal health pre- and postnatally including gestational diabetes, metabolic syndrome, obesity and postpartum depression
- To identify gene-environmental interaction affecting fetal growth and child health
- To assess the prevalence of nutritional, environmental, biological, and psychosocial exposures in the prenatal period and early childhood
- To examine the impact of these exposures on health-related outcomes such as birth outcomes, neurodevelopment, obesity, asthma and allergies that appear in early life and is likely to perpetuate into late childhood or adulthood
- To apply new innovative technology platforms "omics" to transfer knowledge from bench side to population side
- To provide valuable evidence-based data to policy makers so as to design effective policies that improve health

This is a multidisciplinary five year cohort study that is developed by the Ministry of Public Health in Qatar (MOPH-Q) in collaboration with various national research institutions, such as Primary Health Care Centers, Qatar University, Weill Cornell Medical Center-Q, Qatar Biomedical Research Institute, Qatar BioBank, Qatar Environmental and Energy Research Institute, Texas A & M University-Q, Sidra Medical Center, Hamad Medical Corporations, and international collaboration with the Barcelona Institute for Global Health (ISGlobal) in Spain.

### Ethical approval

The MOPH-Q has provided local Institutional Review Boards (IRBs) with national guidelines for overseeing research studies that involve vulnerable subjects such as pregnant women and children in order to maintain the safety and wellbeing of these participants. Large cohorts that involve collection of biological samples and handle genetic information present emerging ethical and legal issues including, but not limited to, privacy, psychological, legal, social, consent, etc. Many of these risks involve the potential for a breach of confidential information. Therefore, decreasing the risks of a breach of confidentiality reduces the risk of subsequent psychological, privacy, legal, and social harms. In order to mitigate those risks while conducting this cohort study, three main approaches are currently in place to secure collected information: 1- physical procedures that include locked cabinets, locked offices, and housing of database servers in rooms with tightly controlled access. 2- Technical procedures that include password protection, encryption, and firewalls. These procedures are designed to limit access to confidential information to those individuals or computer programs that have been granted access rights. 3- Administrative procedures such as procedures for training research staff, confidentiality agreements, and certificates of confidentiality. In addition, continuous training sessions is provided to the research team ensuring that every step in participation is understood (e.g., translating documents to appropriate languages). Trained staff assesses the informed consent and make sure that the participants have understood the purpose of the study, including the duration of participation, procedures/methods, risks and benefits, and the terms of confidentiality. It is clearly stated within the consent form and the information flyer that their participation is voluntary at every point in the project.

The study has been reviewed and ethically approved by the Institutional Review Board (IRB) of the Qatar BioBank Ethics Committee (Protocol ID: Full Board/2017/RES-ACC-0067/0019).

### Study population

**Fig. 1:**
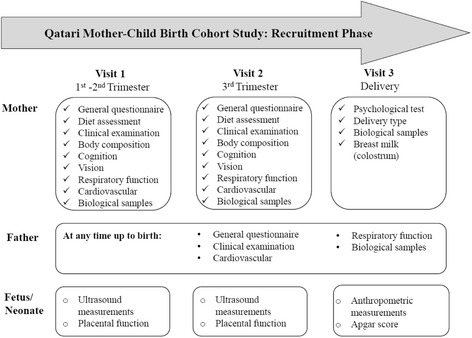
QBiC follow-up visits during pregnancy

Eligible participants for this study are all pregnant women who have lived in Doha for at least 15 years, and will give birth in Qatar within the 2 year period (anticipated number: 3000 families) of recruitment along with their husbands. This is the first phase of the study where the pregnant Qatari women and their husbands are being contacted before the 15th week of gestation at the Primary Health Care Centers (PHCC) in Doha, Qatar. Study participants need to be residents in the study area, older than 16 years, with no communication handicap. The consented participants are interviewed to obtain information on several sociodemographic and lifestyle factors and maternal characteristics are assessed based on anthropometric measurements, spirometry, blood pressure and other clinical measurements. Pregnant women are invited to provide blood, urine, saliva, hair and feces samples throughout their pregnancy, as well as cord blood and tissue, placental tissue, colostrum and breast milk at delivery, according to the scheduled visits (Fig. 1).

### Recruitment and data collection

Research assistants contact pregnant women at the PHCC during their first scheduled major ultrasound test, and kindly invite them to participate in the QBiC study. Those interested to participate are scheduled for a visit at Qatar Biobank where they have the opportunity to discuss the study in depth with a research member and provide a written consent form. Consented participants, are invited to complete a baseline questionnaire, via face to face interview, including sociodemographic and lifestyle characteristics, and environmental exposures. Table 1 provides an overview of assessments and data that are collected at recruitment phase up to delivery for the pregnant women, the fathers and the fetus. Dietary habits of the women are assessed with the use of a food frequency questionnaire (FFQ) and 24-h recalls. Research assistants also assess anthropometric measurements of the participants including height, weight, waist circumference and hip circumference, bio-impedance analysis, cognitive function test, grip strength, vision, cardiovascular system (i.e. blood pressure, electrocardiogram, arterial stiffness, carotid ultrasound), respiratory function (by spirometry) and fetus ultrasound measurements.

**Table 1:** Data planned to be collected at recruitment phase up to delivery

Follow-up	Questionnaires	Clinical examination	Ultrasound measurements	Biological samples
Mother				
12–15 weeks	General FFQ 24 h recall	Anthropometric characteristics Skinfolds Bioimpedance SCORAD test Metabolic syndrome Blood pressure Spirometry Thyroid malfunction Gestational diabetes	Placental function: resistance indices of the uterine and umbilical arteries	Blood Urine Saliva Feces
32 weeks	General FFQ 24 h recall	Anthropometric characteristics Bioimpedance Skinfolds Blood pressure Spirometry Thyroid malfunction Gestational diabetes	Placental function: resistance indices of the uterine and umbilical arteries	Blood Urine Saliva Feces
Delivery	Psychological	Gestational diabetes, preeclampsia, hypertension		
Father				
1st Visit	General	Anthropometric characteristics Bioimpedance Skinfolds Blood pressure		Blood Urine Saliva Feces Semen
Fetus				
12–15 weeks			CRL, HC, BPD, FL, AC	
32 weeks			HC, BPD, FL, AC Organ development: brain, heart, kidneys, blood flow	
Birth		Clinical records: induced abortion fetal or perinatal loss, caesarean	Sex, weight, length, HC, AC, Apgar score	Cord blood Cord tissue Placenta Meconium Colostrum Breast milk Hair

*Abbreviations*: *FFQ* Food Frequency Questionnaire, *CRL* Crown-rump length, *HC* head circumference, *BPD* biparietal diameter, *FL* femur length, *AC* Abdominal circumference

During the third trimester, pregnant women will be approached again for a follow up visit similar to the first one where maternal stress will be also assessed via questionnaire. Pregnancy-related complications and fetal ultrasound measurements will be obtained by the medical records of the participants. At birth, research assistants will clinically examine the infant (weight, length, anogenital distance, head circumference) and collect information on the type of delivery, infant sex and gestational age. Pregnant women will be invited to provide blood and urine samples at the scheduled visits during pregnancy, as well as cord blood and colostrum at delivery. Fathers who accompany their wife will also be invited to participate in the study. Once they agree they will be invited for a visit at Qatar Biobank where they will be informed about the study by trained personnel. Consented participants will be asked to complete a face to face questionnaire regarding their lifestyle and medical history and to provide biological samples (Blood, urine, saliva, feces and semen). All clinical visits of the participants will be located at Qatar Biobank.

The biochemical profile of the pregnant women, their husbands and their children will be assessed including total cholesterol, high-density lipoprotein (HDL), low-density lipoprotein (LDL), glucose, insulin, thyroid hormones and antibodies, leptin, adiponectin, C - reactive protein (CRP), inflammatory biomarkers. ‘Omic’ analyses will be conducted in mothers, fathers and children with main focus on the identification of early markers of maternal health effects and reproductive outcomes and on the evaluation of gene-environment interactions.

### Withdrawal

It is a fundamental right of the participant to withdraw from the study at any time without explanation. Pregnant women and their husbands are informed of their right to withdraw at any time and whether they wish their samples collected up to that point to be destroyed and their personal data erased from the databases. However, samples and data generated from their samples that have already been provided to other researchers or research centers or are placed in a formal research database cannot be withdrawn.

### Data warehouse and biological samples

The enormous amount of data expected to be generated will be incorporated in order to provide an integrated data warehouse at the Qatar biobank (QBB). A secure and private password will be required in order to proceed with the data exchange. Access to these protected data will be limited to the project researchers. Each participant of the study is allocated with a unique study number and this will be used to identify its data throughout the study. Personal details such as name, surname or date of birth will never appear on the samples, and the generated data will be untraceable in order to protect confidential individual information. The QBB will be responsible for the biological samples collected at the different time points, for the biosample processing and their provision to other collaborators [20]. At a later stage a common database will be created including all available information such as nutritional, lifestyle, environmental exposures, sociodemographic characteristics, biomarkers and clinical data. All samples will be stored in ultra-low temperature freezers (−80 °C).

### Statistical considerations

Additional challenges related to evidence-based epidemiological studies include the investigation of multiple exposures and outcomes. Most commonly, studies examine the effect of a single exposure to a single outcome, whereas pregnant women and children are exposed to multiple hazards. In the current study, the burden of disease estimates in relation to environmental, lifestyle, nutritional exposures will be obtained by integrating exposure, biomarker and outcome data, along with the corresponding risk estimates and exposure-response data. Initially, one exposure-one health outcome assessments will be conducted followed by more advanced models that will be developed incorporating genetic susceptibility to capture the observed complexity and try to integrate risk and benefits in a more holistic model through analyses from sources to health outcomes. Given the complexity of the dataset that will be originated from this study, it is expected that an interplay between a priori biological knowledge and ‘unsupervised’ statistical methods will be the most fruitful approach to discovery. For example, pathway analysis will exploit both existing databases and already developed statistical tools (such as Factor Analysis and Latent Class Analysis) to identify patterns [21, 22]. Another challenge for the present study will be to have complete datasets of measurements for all mother-children pairs instead of limited subsets of measurements for a subsample of the total participants, related to specific hypotheses. In these cases, the use of imputation techniques, where applicable, will be effective in reducing the bias resulting from missing values [23]. This will provide researchers the flexibility to investigate the effect of a wider range of exposures and their interaction.

## Discussion

As with all longitudinal cohort studies a major concern is to select the study participants from the source population and avoid as much as possible any selection bias arising from the procedures followed at the recruitment phase and/or during the procedure of retaining them in the study [24]. In order to address these biases it will be necessary to regularly examine the differences in characteristics between participants and non-participants according to the research questions. At recruitment, individuals who volunteer to participate in a study have generally different baseline characteristics than the non-responders. Sample attrition is (withdrawals or loss to follow up) a very common source of bias in cohort studies and it will be a big challenge for QBiC study to keep the families enrolled for long term follow ups. Regular contact with the families, personal invitations, survey publicity and consecutive newsletters will be needed in order to retain the study participants and overcome attrition bias. Additional effort will also be required by the researchers to maintain representativeness throughout the study.

One of the main strengths of the QBiC study is that it will be the first large scale mother-child cohort study in the Middle East studying the gene-environment interactions associated with health impact. It is a unique, national, evidence-based, epidemiological study designed to assess global viewing on how various types of environmental exposures co-exist and jointly with individual genome variability impact on health during critical developmental periods. This multi-disciplinary project will assess the synergetic role of environmental exposure and genetic factors in the development of chronic disease and monitor woman and child health and/or obstetric characteristics with high prevalence in the state of Qatar. Novel tools and methods will be implemented to obtain estimates of individual environmental exposures including outdoor and indoor air pollution, for study participants.

Proper recruitment strategy of the study will provide excellent opportunity to researchers to bring together several key elements in terms of sample size, sampling strategy, exposure measures and phenotyping characterization. This way the research team will be able to more efficiently address future challenges regarding the following phases of the study. The rapid change in biotechnology indicates that innovative approaches will have to be continuously applied in QBiC study. The harmonization of data within the study will allow to develop future project collaborations with other European and International birth cohorts. The need for a broader geographical representation of the existing or new birth cohorts constitutes QBiC important, as the unique birth cohort study in Middle East. It will provide information to the wider scientific community and through joint governance and clear data access policies aims to participate to interdisciplinary research and share of the longitudinal data, and thus contribute to science and public health agenda.

## Abbreviations

CRP: C - reactive protein
DOHaD: Developmental Origins Of Health And Disease
FFQ: Food Frequency Questionnaire
GCC: Gulf Cooperative Council
HDL: High-Density Lipoprotein
IRBs: Institutional Review Boards
ISGlobal: Barcelona Institute For Global Health
LDL: Low-Density Lipoprotein
PHCC: Primary Health Care Centers
QBB: Qatar Biobank
QBiC: Qatari Birth Cohort Study
STEPS: STEPwise Approach To Surveillance
WHO: World Health Organization
